# Supplementary material for: Integrative Transcriptomic and Phytohormonal Analyses Provide Insights into the Cold Injury Recovery Mechanisms of Tea Leaves
Source: Plants (Basel). 2022 Oct 18;11(20):2751. doi: 10.3390/plants11202751 (PMC9610371; doi:10.3390/plants11202751)
Supplement: Supplementary file 1 [file plants-11-02751-s001.zip › Table S1.pdf]

Table S1. Statistics on the RNA-Seq data.

Sample	Raw data (bp)	Clean data (bp)	Clean reads	Genes matched to reference genome (%)
Row 2_1	6724865100	6676321645	44761522	29321 (86.41%)
Row 2_2	6206443800	6169568604	41308512	24690 (72.76%)
Row 2_3	6916097400	6880030586	46034736	24361 (71.79%)
Row 5_1	7842801300	7795403591	52203746	24496 (72.19%)
Row 5_2	6475807200	6438598476	43104232	25164 (74.16%)
Row 5_3	5720299500	5684113075	38065882	24938 (73.49%)
Row 8_1	7418668200	7359537936	49364428	24664 (72.69%)
Row 8_2	6053926800	6008380348	40275690	25173 (74.19%)
Row 8_3	7301307900	7255449941	48588656	24935 (73.49%)
Row 11_1	7599596400	7555110233	50583830	25063 (73.86%)
Row 11_2	7371006600	7325790371	49063844	25091 (73.94%)
Row 11_3	5920353900	5881491265	39409710	25074 (73.89%)
