# Supplementary material for: Integrative Transcriptomic and Phytohormonal Analyses Provide Insights into the Cold Injury Recovery Mechanisms of Tea Leaves
Source: Plants (Basel). 2022 Oct 18;11(20):2751. doi: 10.3390/plants11202751 (PMC9610371; doi:10.3390/plants11202751)
Supplement: Supplementary file 1 [file plants-11-02751-s001.zip › Table S5.pdf]

Table S5. The primer sequences for qPCR.

Gene		primer (5′→3′)
β-actin	F	CTTCCTCATGCTATCCTCCGTCTT
	R	ATTTCCCGTTCAGCAGTGGTG
PAL	F	ACGACAACCCCTTGATCGAC
	R	TTGATGCCAAAGCCAGCCTA
4CL	F	TTCATGCGGAACTGTGGTCA
	R	TGGAGCCAACCATCCACATC
CHS	F	GCAGGACATGGTTGTGGTTG
	R	TTGACTGATGGGCGAAGACC
LAR	F	GCCTACGTACCTTCTCGTCC
	R	CAACGTCATGCCCAAACTCC
F3’H	F	CACCCATCAACCCCACTCTC
	R	GCCACCAGGTAGGAATCGTT
DFR	F	TGCCAGTTGTGTCGTTCTC
	R	AGCAAACCCTTCTCTCTGC
C4H	F	TCAAGGACACGAGGTTGCAG
	R	TGGGTGGTTGACGAGTTCTG
ANS	F	AACAAGCGAGTACGCAAAGC
	R	TGAAGCTCTTCCATGCCTCC
FLS	F	CCCTCGGAGTTGAACCTCAC
	R	ACGACAAACACAGCCCAAGA
