# Supplementary figures and images for: Integrative Transcriptomic and Phytohormonal Analyses Provide Insights into the Cold Injury Recovery Mechanisms of Tea Leaves
Source: Plants (Basel). 2022 Oct 18;11(20):2751. doi: 10.3390/plants11202751 (PMC9610371; doi:10.3390/plants11202751)

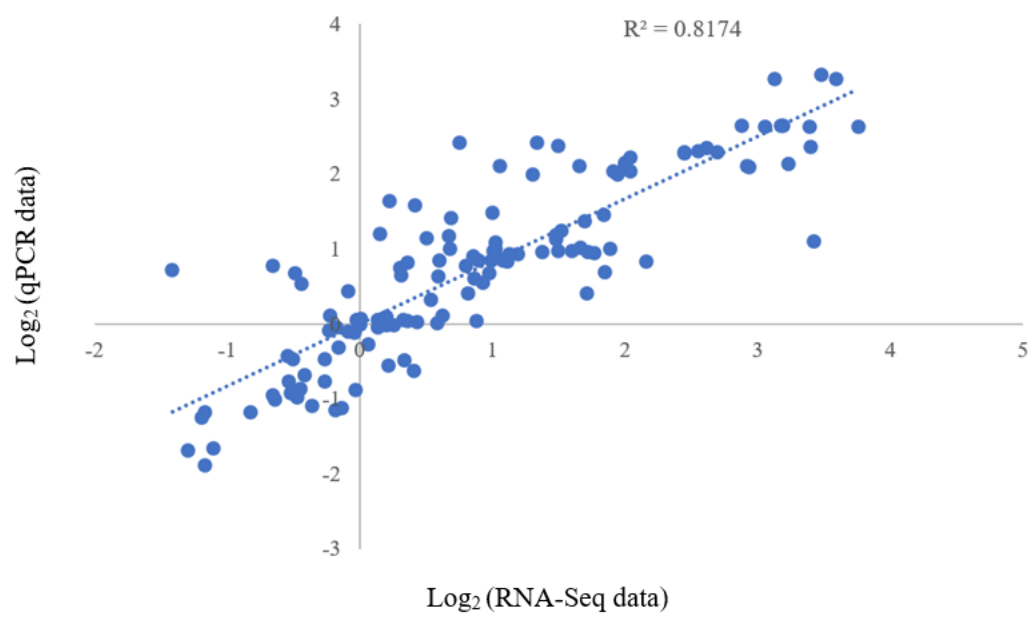

**Figure S1.** The correlation between RNA-Seq result and qPCR data

Supplement: Supplementary file 1 [file plants-11-02751-s001.zip › Figure S1.pdf]
